# Inequality in mortality according to regional deprivation during the COVID-19 pandemic

**DOI:** 10.4178/epih.e2025022

**Published:** 2025-04-29

**Authors:** Min Hui Moon, Young Gyu Ko, Min Hyeok Choi

**Affiliations:** 1 Department of Preventive and Occupational and Environmental Medicine, Pusan National University School of Medicine, Yangsan, Korea; 2 Office of Public Healthcare Service, Pusan National University Yangsan Hospital, Yangsan, Korea; 3 Busan Public Health Policy Institute, Busan, Korea

**Keywords:** COVID-19, Mortality, Health inequalities, Socioeconomic factors

## Abstract

**OBJECTIVES:**

Vulnerability to coronavirus disease 2019 (COVID-19) is significantly greater in regions with lower socioeconomic status (SES). However, detailed analyses of regional socioeconomic disparities have rarely been conducted in Korea. This study aimed to identify and compare mortality inequalities associated with regional SES across different areas of Korea during the COVID-19 pandemic.

**METHODS:**

Using cause-of-death statistics from 2020 to 2022, we calculated age-standardized mortality rates (ASMRs) for total mortality, COVID-19 mortality, and pneumonia mortality. The SES of each region was assessed using the regional deprivation index. Additionally, we calculated the rate difference, rate ratio, slope index of inequality (SII), and relative index of inequality (RII) for each socioeconomic level to examine the extent of mortality inequality and its temporal changes. These analyses were stratified by gender and urban-rural classification.

**RESULTS:**

The total mortality rate, as well as COVID-19-specific and pneumonia-specific mortality rates, increased during the COVID-19 pandemic. The ASMR for COVID-19 was higher in rural areas (ASMR, 27.79), which have lower healthcare accessibility, compared to urban areas (ASMR, 26.63). However, mortality inequality indicators for COVID-19 were more pronounced in urban areas compared to rural areas (SII: urban, 2.72; rural, -0.05, RII: urban, 0.10; rural, 0.00). Notably, significant inequalities were observed among men residing in urban areas.

**CONCLUSIONS:**

In disaster situations such as the COVID-19 pandemic, it is essential to implement strategies aimed at reducing overall mortality rates and addressing regional socioeconomic inequalities.

## GRAPHICAL ABSTRACT

[Fig f2-epih-47-e2025022]

## Key Message

During the COVID-19 pandemic, regional socioeconomic disparities significantly influenced mortality in Korea. Mortality rates were higher in deprived rural areas, while relative inequalities were more evident in urban men. Tailored policies addressing both absolute and relative inequalities are essential to ensure equitable healthcare access in future crises.

## INTRODUCTION

The coronavirus disease 2019 (COVID-19) pandemic has had unprecedented impacts on physical and mental health, mortality, and healthcare systems worldwide. Since its onset, more than 687 million confirmed cases and approximately 6.9 million deaths have been officially recorded over a span of 3 years and 4 months, leading up to the declaration by the 14th World Health Organization International Health Regulations Emergency Committee that ended the public health emergency of international concern [1]. In Korea, approximately 34.6 million cumulative confirmed cases and around 35,000 deaths have been reported, with a notable increase in the mortality rate as the pandemic progressed [2].

Mortality-related indicators, which are essential metrics for evaluating COVID-19’s overall societal impact, are used to measure not only the direct effects of COVID-19 but also health inequalities [3-9]. For instance, it has been documented that groups characterized by lower education levels, lower incomes, or unstable employment statuses face a higher risk of COVID-19 infection and experience higher rates of severe illness and mortality [10,11]. Mortality inequalities related to COVID-19 may manifest due to individual factors, including gender, age, and socioeconomic status (SES) [10,12,13], as well as regional factors such as residential area and environmental conditions [11,14-17]. KC et al. [15] observed higher incidence and mortality rates in regions with lower SES during the COVID-19 pandemic, emphasizing that regional living conditions and socioeconomic contexts significantly influence individual health outcomes related to COVID-19. Barnard et al. [16] identified notable disparities in excess mortality associated with varying regional deprivation levels in the United Kingdom during the COVID-19 pandemic.

Most studies examining COVID-19-related mortality inequalities in Korea have thus far primarily focused on individual-level SES. Although international studies have analyzed the effects of regional socioeconomic disparities, such research remains limited within Korea. Considering this gap, the current study aims to complement and extend existing research in 3 specific ways. First, this study evaluates not only COVID-19 mortality but also mortality due to all causes and respiratory diseases, conditions that represent similar burdens of disease influenced by regional deprivation during the pandemic. During the pandemic, healthcare service utilization declined [18], and the healthcare system experienced considerable strain due to its focus on managing COVID-19 patients [19]. Some studies have indicated that the reduction in healthcare utilization was more pronounced in urban healthcare facilities [20]. This finding suggests potential regional disparities in COVID-19 incidence rates and implies that inadequate healthcare resources available to non-COVID-19 patients could have contributed to increased mortality rates. By assessing mortality from COVID-19, pneumonia, and overall mortality, we can examine the implications for healthcare systems and inform policy responses from diverse perspectives.

Second, numerous studies have reported that large-scale disasters, such as earthquakes, the Spanish flu, and H1N1, have varying impacts across socioeconomic strata [10,11,13,14,21,22]. Catastrophic events generate comprehensive consequences, including direct damage, socioeconomic impacts such as income reduction and unemployment, and health problems like disease, anxiety, and depression [13,21,23]. The COVID-19 pandemic has similarly demonstrated how regional socioeconomic deprivation exacerbates health disparities and leads to higher mortality rates in socioeconomically disadvantaged areas during health crises [11,24,25]. Despite the pressing need for evidence on how regional deprivation levels influence mortality inequality during the COVID-19 pandemic in Korea, research addressing this issue remains sparse.

Third, gender differences have been observed during the COVID-19 pandemic, with men exhibiting higher disease severity and fatality rates compared to women [26-29]. Differences have also emerged between urban and rural areas [30,31]. In urban areas, COVID-19 incidence rates were higher, whereas rural areas experienced relatively lower incidence rates but higher mortality rates [31]. This pattern has been attributed to the higher proportion of elderly individuals with underlying health conditions and lower healthcare accessibility in rural areas compared to urban regions [30]. Based on previous research, it is necessary to analyze and identify variations in mortality inequalities at the regional level by stratifying data according to gender and urbanization.

This study was undertaken to clarify how mortality rates have changed according to regional deprivation levels during the COVID-19 pandemic crisis and to identify regional mortality inequalities. Such findings will contribute more robust evidence to support effective crisis response policies. Specifically, the objectives of this study were to (1) determine age-standardized mortality rates (ASMRs) for all-cause mortality, pneumonia mortality, and COVID-19 mortality based on regional deprivation levels during the COVID-19 pandemic period (2020-2022); (2) quantify the magnitude of inequalities in all-cause mortality, pneumonia mortality, and COVID-19 mortality associated with regional deprivation levels during the pandemic using inequality measurement indicators; and (3) assess and observe changes in the magnitude of inequalities for all-cause mortality, pneumonia mortality, and COVID-19 mortality according to regional deprivation levels, stratified by gender and urban-rural classification.

## MATERIALS AND METHODS

### Data

This study utilized raw data from the Cause of Death Statistics and the Resident Registration Population provided by the National Statistical Office for the years 2020 through 2022. Such statistical data are appropriate for understanding the scale and structure of mortality causes across the entire population. They include essential information related to deaths, such as gender, age, date of death, residential address of the deceased (administrative district code), and cause of death. Causes of death were classified according to the International Classification of Diseases, 10th revision (ICD-10). The final analysis included 995,515 deceased individuals, after excluding 52 individuals with missing critical information such as cause of death or age.

### Mortality

In this study, overall mortality (total deaths) for each year (2020, 2021, and 2022), mortality due to COVID-19 (ICD-10 codes U071, U072, U109), and mortality from pneumonia (ICD-10 codes J12-J18)—a similar respiratory disease—were measured and compared. The mid-year resident population for each year was used as the denominator for calculating mortality (Supplementary Materials 1-3). To facilitate comparison of mortality levels among groups with different population structures, we calculated the ASMR, an indicator that accounts for differences in age structure. The standard population for ASMR calculations was based on the estimated Korean population from 2005.

### Deprivation index

The deprivation index is a composite measure that represents the extent of material and social deprivation within specific regions. It was constructed using several standardized and weighted variables [32] and is commonly utilized in studies examining health inequalities by regional socioeconomic levels [33-36].

This study’s deprivation index was calculated using data from the 2020 Korean Census, which sampled 20% of the population, and regional data from the Micro Data Integrated Service 2020. The index was computed for all 250 districts (*gu* and *gun*) across Korea. To create this index, 10 variables were selected through factor analysis using varimax rotation. The scores for each factor were standardized through a z-transformation and then summed to yield the final deprivation index. The 10 variables included were: (1) proportion of households with low SES (based on the household head), (2) homeownership rate, (3) proportion of households living in poor housing conditions (with at least 1 issue), (4) proportion of the population with less than a high school education, (5) proportion of households without a car, (6) divorce/widowhood rate, (7) proportion of single-person households, (8) proportion of women-headed households, (9) proportion of older residents, and (10) proportion of apartment households. Higher deprivation scores indicate greater socioeconomic vulnerability. Regions were classified into 3 categories based on their deprivation index scores: lowest, middle, and highest deprivation levels.

### Inequality measures and statistical analysis

In this study, multiple methods were employed to assess mortality according to regional deprivation levels. These included pairwise comparison measurements (rate difference [RD] and rate ratio [RR]) and regression-based inequality measures, specifically the slope index of inequality (SII) and the relative index of inequality (RII), which account for population proportions at each socioeconomic level. RD represents the absolute difference in mortality rates between the lowest and highest deprivation levels, while RR represents the mortality RR between these levels. The SII is a regression-based measure of inequality that assesses disparities across socioeconomic groups ranked by deprivation level. To calculate SII, regions were ordered from lowest to highest deprivation. Each deprivation category corresponds to a specific range in the cumulative population distribution, and the midpoint of each range was assigned to represent that group. Mortality rates were plotted against these midpoints, and a regression line was fitted; the slope of this line corresponds to the SII. The RII measures the relative magnitude of this absolute difference by dividing SII by the overall population’s average mortality rate [37]. For instance, if the SII is 1, it indicates that the health level improves by 1 unit with every incremental increase in SES. Conversely, the RII demonstrates how significant the change in health level is relative to the average health level of the entire population. Although SII and RII yield more stable estimates with an increased number of socioeconomic position groups, they are effective for assessing health inequalities even with 3 or more categories, as they adjust for the underlying population structure [38-41]. In this study, both absolute measures (RD, SII) and relative measures (RR, RII) were applied to evaluate inequalities between extreme deprivation groups and to assess the broader socioeconomic gradient in health disparities. All inequality indicators were calculated based on the cause of death, stratified by gender and urban-rural (*si-gun-gu*) classifications for the years 2020 to 2022. Statistical analyses were performed using SAS version 9.4 (SAS Institute Inc., Cary, NC, USA) and R version 4.2.2 (R Foundation for Statistical Computing, Vienna, Austria).

### Ethics statement

The study protocol was approved by Pusan National University Hospital (No. 55-2023-016).

## RESULTS

We examined the ASMR, RD, and RR for all-cause mortality, pneumonia mortality, and COVID-19 mortality during the study period. As shown in Table 1, the ASMR for all-cause mortality increased from 315.72 (95% confidence interval [CI], 314.52 to 316.92) in 2020 to 354.44 (95% CI, 353.21 to 355.67) in 2022. Similarly, the ASMR for COVID-19 markedly increased from 0.88 (95% CI, 0.82 to 0.94) in 2020 to 26.72 (95% CI, 26.41 to 27.02) in 2022. The ASMR for pneumonia displayed a slight decline from 19.78 (95% CI, 19.52 to 20.05) in 2020 to 19.58 (95% CI, 19.32 to 19.84) in 2021, followed by an increase to 22.03 (95% CI, 21.75 to 22.30) in 2022. The ASMRs for all-cause, pneumonia, and COVID-19 mortality were consistently higher among men than among women.

For all-cause mortality and pneumonia, mortality rates increased with higher deprivation levels, a pattern observed in both men and women. For COVID-19 mortality, the highest deprivation levels were associated with increased mortality rates in 2022 when deaths significantly increased. However, in 2020 and 2021, when death counts were comparatively low, inequalities in ASMR by deprivation level were inconsistent. The RD for all-cause ASMR increased slightly from 43.81 (95% CI, 38.91 to 48.71) in 2020 to 47.63 (95% CI, 42.54 to 52.72) in 2022, whereas the RR remained relatively stable. In contrast, the RD for pneumonia ASMR decreased from 3.19 (95% CI, 2.15 to 4.23) in 2020 to 2.87 (95% CI, 1.80 to 3.93) in 2022, and the RR also showed a slight reduction from 1.17 (95% CI, 1.11 to 1.23) to 1.13 (95% CI, 1.08 to 1.19). The most substantial increases in the RD and RR occurred in COVID-19 ASMR. While no significant inequality was observed in COVID-19 mortality in 2020 and 2021, the RD shifted direction in 2022 from -0.05 (95% CI, -0.27 to 0.18) to 1.69 (95% CI, 0.48 to 2.90), and the RR exceeded 1.00 in 2022, reaching 1.06 (95% CI, 1.02 to 1.11). RD and RR values for all-cause mortality, pneumonia, and COVID-19 were consistently higher among men than women.

**Figure 1.**
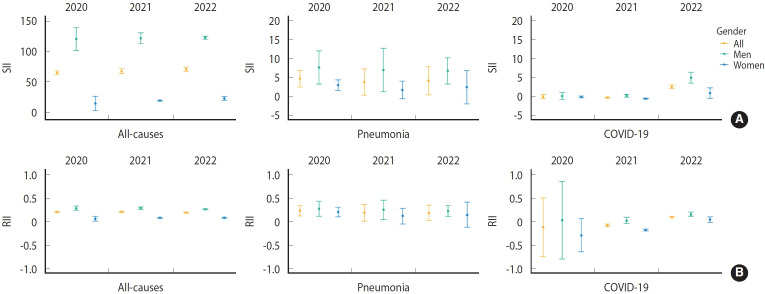
The (A) slope index of inequality (SII) and (B) relative index of inequality (RII) for all-cause, pneumonia-specific, and coronavirus disease 2019 (COVID-19)-specific mortality according to deprivation level from 2020 to 2022.

Figure 1 shows the SII and RII for all-cause mortality, pneumonia, and COVID-19 ASMRs by year. Throughout the study period, no significant changes were noted in SII or RII for all-cause or pneumonia mortality. However, the SII for COVID-19 mortality significantly increased from -0.11 (95% CI, -0.66 to 0.44) in 2020 to 2.52 (95% CI, 2.06 to 2.98) in 2022, showing a reversal in direction (sign). Similarly, the RII increased steadily from -0.12 (95% CI, -0.74 to 0.50) in 2020 to 0.09 (95% CI, 0.08 to 0.11) in 2022, also reversing its sign.

The SII and RII for all-cause mortality, pneumonia, and COVID-19 were higher among men compared to women. Specifically, in 2022, age-standardized COVID-19 mortality rates exhibited increased absolute and relative inequalities in both genders, with notably higher inequalities observed among men (SII for men: 4.88; 95% CI, 3.44 to 6.33; SII for women: 0.83; 95% CI, -0.56 to 2.21; RII for men: 0.15; 95% CI, 0.11 to 0.20; RII for women: 0.04; 95% CI, -0.02 to 0.10).

Tables 2-4 present the calculated ASMRs for all-cause mortality (Table 2), pneumonia mortality (Table 3), and COVID-19 mortality (Table 4), stratified by deprivation level, gender, and urban-rural classification during the study period. Compared with 2020, mortality rates for all causes, pneumonia, and COVID-19 increased in both urban and rural areas by 2022. Except for COVID-19 mortality rates in 2020 and 2021, mortality rates were consistently higher in rural areas compared to urban areas across all categories.

### All-cause mortality

The RD for all-cause mortality by deprivation level increased in rural areas from 32.98 (95% CI, 15.54 to 50.42) in 2020 to 52.42 (95% CI, 34.85 to 69.98) in 2022. The RD among men increased in urban areas but decreased in rural areas, whereas the RD among women showed the opposite trend, decreasing in urban areas and increasing in rural areas. The RR for men and women either slightly decreased or remained stable in urban areas, whereas in rural areas, the RR decreased among men and increased among women. The SII was higher among men than women in both urban and rural regions, with an increasing trend among urban men over time. The RII values were similar in urban and rural areas; however, no observable inequality was detected among rural women.

### Pneumonia

The RD and RR for pneumonia mortality by deprivation level gradually decreased from 2020 to 2022 in both urban and rural regions. The RD was notably higher in rural areas than in urban areas and larger for men than women. Both the SII and RII declined across all groups. Among urban women, higher deprivation levels were associated with lower pneumonia mortality rates, suggesting an absence of observable absolute or relative inequalities in this subgroup.

### Coronavirus disease 2019

The COVID-19 mortality rate by deprivation level dramatically increased across all groups in 2022, irrespective of gender or region. In urban areas, the RD was higher for men (3.45; 95% CI, 1.01 to 5.88) than for women (0.65; 95% CI, -1.10 to 2.41). RR values were similar for both genders. For COVID-19 ASMR in urban areas by deprivation level, men had a significantly higher SII (4.84; 95% CI, 2.20 to 7.47) and RII (0.15; 95% CI, 0.07 to 0.23) than women (SII: 1.04; 95% CI, -0.12 to 2.21; RII: 0.05; 95% CI, -0.01 to 0.10). Conversely, in rural areas, the RR, SII, and RII for COVID-19 mortality by deprivation level were higher among women than among men, excluding RD. Nevertheless, the magnitude of inequality (RD, RR, SII, and RII) remained greater in urban areas than in rural areas.

## DISCUSSION

This study examined changes in ASMR for all causes, pneumonia, and COVID-19 according to regional socioeconomic levels during the COVID-19 pandemic period (2020-2022). Additionally, trends in mortality inequality were analyzed. The primary objective was to provide an evidence-based foundation for developing policies aimed at mitigating health inequalities that arise during large-scale infectious disease pandemics.

From 2020 to 2022, the ASMR for all-cause mortality, pneumonia, and COVID-19 consistently increased. Relative inequalities (RR and RII) for all-cause mortality by regional deprivation level remained stable, whereas absolute inequalities (RD and SII) increased. Pneumonia mortality inequality by deprivation level showed either a slight decrease or minimal changes. Conversely, COVID-19 mortality inequalities either reversed or increased. In the early phase of the pandemic in Korea, COVID-19 affected only specific regions, resulting in ambiguous or reversed regional inequalities. However, following the domestic introduction of the Omicron variant in 2021 and the subsequent widespread outbreak, clear inequalities in COVID-19 mortality according to regional SES became evident [42]. These findings align with McGowan & Bambra [11], who found that COVID-19 intensified pre-existing chronic health inequalities and highlighted mortality disparities in socioeconomically disadvantaged areas.

Examining the ASMR by regional deprivation levels, differentiated by urban and rural areas, showed that in 2020 and 2021, mortality rates from all causes and pneumonia were higher in rural areas than urban areas, whereas COVID-19 mortality showed the opposite trend. However, in 2022, similar to all-cause and pneumonia mortality, COVID-19 mortality rates were higher in rural regions compared to urban regions. The higher mortality in rural areas can be attributed to multiple factors, including an aging population, limited access to healthcare, and a greater prevalence of preventable diseases. Access to healthcare services is a critical determinant of health outcomes, including mortality [43]. In Korea, rural medical institutions provide 122,310 hospital beds, approximately 23% of the total available in urban medical facilities [44]. The number of doctors per 1,000 individuals averages 3.41 in urban areas and only 1.98 in rural areas, indicating that rural residents often face challenges in accessing timely and appropriate medical care due to shortages of healthcare facilities, providers, and resources [45]. Even during crisis situations such as the COVID-19 pandemic, disparities in healthcare access and availability likely contributed significantly to observed differences in mortality.

Inequality indicators for COVID-19 mortality according to deprivation level either increased or reversed direction in both urban and rural areas. Although COVID-19 mortality rates in 2022 were higher in rural regions, urban areas exhibited greater inequalities. Rural areas generally show higher average deprivation levels (mean±standard deviation, 5.68±4.67) compared to urban areas (mean±standard deviation, -2.77±5.28), suggesting relatively less pronounced inequality in ASMR within rural contexts. However, disparities in deprivation levels among districts within urban areas (interquartile range, 7.54; range, 29.93) were larger than those in rural areas (interquartile range, 6.34; range, 22.55), contributing to the greater inequalities observed in urban environments. Although differences in income and health are generally less distinct in rural settings, disparities between smaller local regions within urban areas are more significant [46]. From the standpoint of deprivation levels, smaller variations between rural districts, combined with overall higher deprivation, may explain why inequalities appear less pronounced overall in rural areas. Thus, rural areas require strategies aimed at reducing overall mortality, whereas urban areas need policies targeting the reduction of regional inequalities.

In terms of gender differences, ASMR due to COVID-19 in 2022 was higher in men than women. This result aligns with findings from other countries, which report higher COVID-19 mortality rates among men compared to women [26-30]. Previous studies in Korea similarly indicated that infection rates were higher among women, while mortality rates were higher among men [29]. Our study also found greater inequalities in COVID-19 mortality associated with regional deprivation among men, particularly among urban men in 2022. High population density in urban settings significantly influences the spread of infectious diseases [30]. Higher men mortality rates may be attributed to pre-existing conditions (e.g., cardiovascular diseases, hypertension, diabetes, chronic respiratory illnesses) and risky behaviors such as smoking, alcohol consumption, and occupational exposures [27]. Additional research is warranted to further explore these issues.

This study had several limitations. First, the case fatality rate of COVID-19 was not considered, potentially affecting mortality analyses. Future research should integrate COVID-19 reporting and investigation data with mortality data to conduct more comprehensive analyses of inequalities related to regional socioeconomic factors. Second, this study did not analyze long-term trends in mortality rates, including COVID-19 mortality. Future studies should examine differences in overall health effects related to mortality according to regional socioeconomic factors across pre-pandemic, pandemic, and post-pandemic periods. Third, we did not directly assess the influence of healthcare supply and accessibility on mortality. Despite these limitations, this study identified significant differences in the magnitude of mortality inequalities according to regional SES during the COVID-19 pandemic. These findings provide essential data to inform policies designed to reduce regional socioeconomic inequalities during disaster situations such as pandemics.

This study aimed to elucidate changes in mortality rates and examine mortality inequalities according to regional socioeconomic levels, confirming that pandemics such as COVID-19 exhibit patterns consistent with established mortality inequalities. The main findings revealed higher overall mortality rates in rural areas, which typically have poorer healthcare access, whereas health inequalities were more pronounced in urban areas. Notably, urban men experienced greater inequality. These findings indicate that strategies to address inequalities during disaster situations like the COVID-19 pandemic should not rely on a single uniform approach. Instead, policymakers should consider a combination of approaches—those that reduce overall mortality and those specifically targeted at mitigating inequalities associated with regional socioeconomic disparities.

## Figures and Tables

**Figure f2-epih-47-e2025022:**
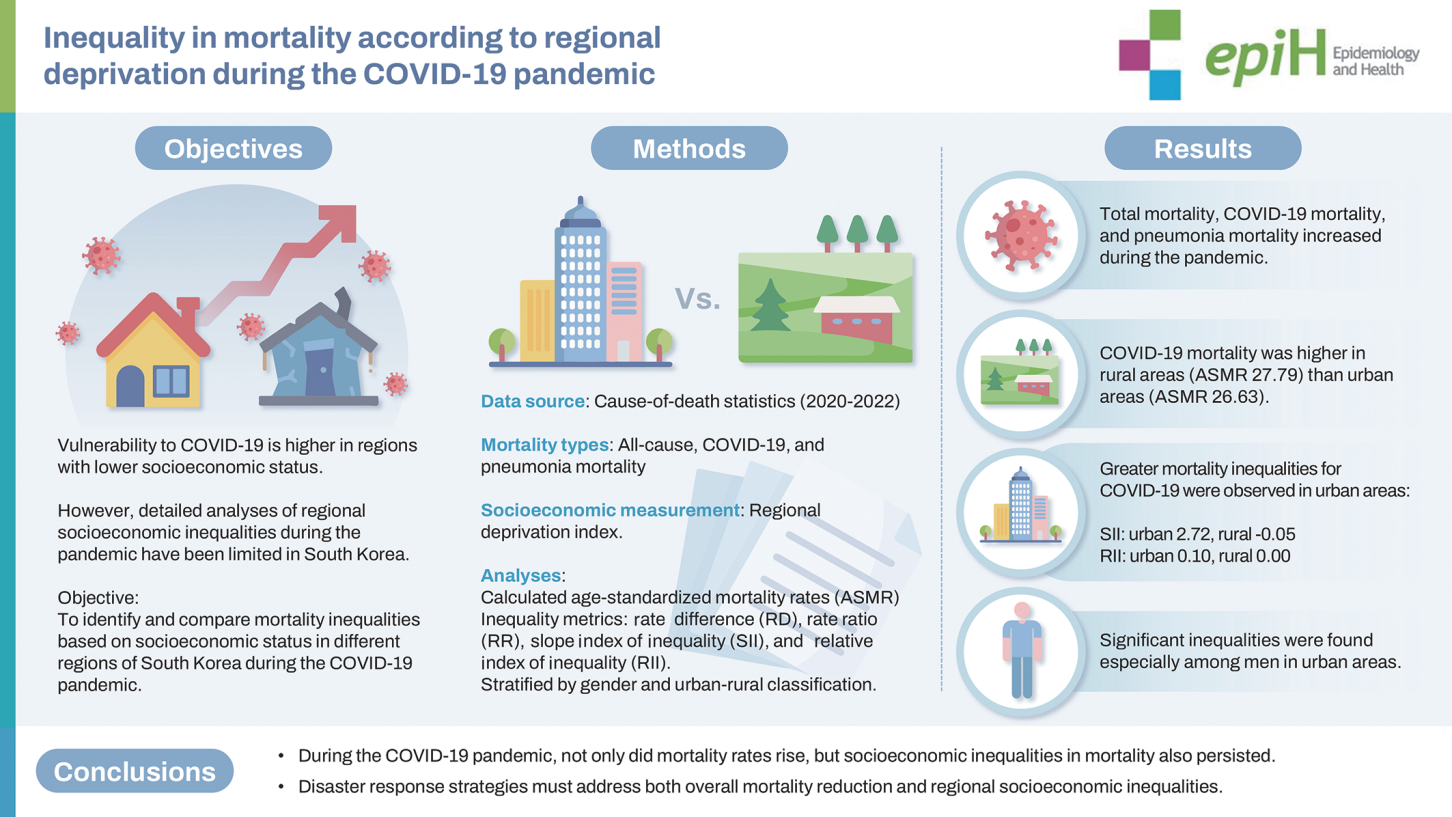


**Table 1.** All-cause, pneumonia-specific, and COVID-19-specific ASMRs, and RD and RR according to deprivation level from 2020 to 2022

Variables	Total			Men			Women		
	2020	2021	2022	2020	2021	2022	2020	2021	2022
All-cause ASMR	315.72 (314.52, 316.92)	317.83 (316.64, 319.02)	354.44 (353.21, 355.67)	409.68 (407.64, 411.72)	410.80 (408.78, 412.81)	448.04 (445.97, 450.10)	235.50 (234.12, 236.88)	237.99 (236.61, 239.36)	272.89 (271.45, 274.32)
Deprivation level									
Lowest	301.42 (299.75, 303.09)	303.38 (301.74, 305.03)	339.10 (337.39, 340.80)	382.84 (380.00, 385.68)	383.18 (380.40, 385.96)	419.54 (416.70, 422.39)	231.79 (229.84, 233.74)	234.59 (232.66, 236.52)	268.79 (266.77, 270.80)
Middle	325.96 (323.83, 328.09)	328.88 (326.75, 331.01)	365.89 (363.69, 368.10)	424.91 (421.30, 428.51)	428.83 (425.24, 432.42)	467.54 (463.85, 471.23)	240.69 (238.23, 243.15)	242.25 (239.80, 244.70)	276.75 (274.19, 279.31)
Highest	345.23 (342.00, 348.45)	349.09 (345.81, 352.37)	386.73 (383.34, 390.11)	464.99 (459.54, 470.44)	465.86 (460.38, 471.34)	502.52 (496.91, 508.13)	240.98 (237.36, 244.60)	247.27 (243.51, 251.02)	284.01 (280.10, 287.93)
Inequality									
RD	43.81 (38.91, 48.71)	45.71 (40.78, 50.64)	47.63 (42.54, 52.72)	82.15 (73.86, 90.44)	82.68 (74.41, 90.94)	82.98 (74.53, 91.43)	9.19 (3.61, 14.76)	12.68 (6.99, 18.36)	15.22 (9.29, 21.16)
RR	1.15 (1.13, 1.16)	1.15 (1.13, 1.17)	1.14 (1.12, 1.16)	1.21 (1.19, 1.24)	1.22 (1.19, 1.24)	1.20 (1.18, 1.22)	1.04 (1.02, 1.06)	1.05 (1.03, 1.08)	1.06 (1.03, 1.08)
Pneumonia-specific ASMR	19.78 (19.52, 20.05)	19.58 (19.32, 19.84)	22.03 (21.75, 22.30)	27.46 (26.97, 27.96)	27.34 (26.86, 27.83)	29.51 (29.02, 30.00)	14.32 (14.03, 14.61)	14.02 (13.74, 14.30)	16.50 (16.19, 16.80)
Deprivation level									
Lowest	18.80 (18.41, 19.19)	18.94 (18.56, 19.32)	21.35 (20.95, 21.74)	25.88 (25.16, 26.60)	26.11 (25.40, 26.81)	28.11 (27.40, 28.82)	13.73 (13.31, 14.16)	13.77 (13.35, 14.19)	16.32 (15.87, 16.77)
Middle	19.94 (19.48, 20.40)	19.39 (18.94, 19.83)	21.87 (21.41, 22.34)	27.53 (26.69, 28.36)	27.14 (26.32, 27.96)	29.73 (28.89, 30.57)	14.43 (13.93, 14.94)	13.74 (13.26, 14.21)	15.99 (15.48, 16.50)
Highest	22.00 (21.35, 22.65)	21.54 (20.91, 22.18)	24.21 (23.54, 24.88)	31.14 (29.95, 32.32)	30.93 (29.74, 32.12)	32.74 (31.55, 33.94)	15.74 (15.01, 16.47)	14.94 (14.29, 15.59)	18.06 (17.28, 18.83)
Inequality									
RD	3.19 (2.15, 4.23)	2.60 (1.58, 3.62)	2.87 (1.80, 3.93)	5.25 (3.35, 7.16)	4.82 (2.93, 6.72)	4.64 (2.73, 6.54)	2.01 (0.85, 3.16)	1.17 (0.10, 2.23)	1.73 (0.51, 2.95)
RR	1.17 (1.11, 1.23)	1.14 (1.08, 1.20)	1.13 (1.08, 1.19)	1.20 (1.13, 1.28)	1.18 (1.11, 1.26)	1.16 (1.09, 1.24)	1.15 (1.06, 1.24)	1.08 (1.01, 1.17)	1.11 (1.03, 1.19)
COVID-19-specific ASMR	0.88 (0.82, 0.94)	4.77 (4.63, 4.90)	26.72 (26.41, 27.02)	1.12 (1.02, 1.22)	6.09 (5.85, 6.32)	31.39 (30.88, 31.91)	0.69 (0.63, 0.76)	3.66 (3.50, 3.81)	22.84 (22.47, 23.21)
Deprivation level									
Lowest	0.97 (0.88, 1.06)	4.88 (4.68, 5.08)	26.12 (25.67, 26.56)	1.20 (1.05, 1.36)	6.02 (5.68, 6.36)	30.34 (29.59, 31.08)	0.77 (0.67, 0.88)	3.88 (3.64, 4.11)	22.60 (22.06, 23.14)
Middle	0.78 (0.68, 0.87)	4.77 (4.53, 5.01)	27.24 (26.71, 27.78)	0.96 (0.80, 1.13)	6.19 (5.78, 6.60)	31.86 (30.98, 32.75)	0.63 (0.52, 0.74)	3.59 (3.31, 3.86)	23.32 (22.66, 23.97)
Highest	0.92 (0.78, 1.06)	4.62 (4.30, 4.95)	27.81 (27.04, 28.57)	1.26 (1.01, 1.50)	6.10 (5.55, 6.65)	33.69 (32.41, 34.96)	0.66 (0.51, 0.80)	3.44 (3.06, 3.82)	23.11 (22.20, 24.03)
Inequality									
RD	-0.05 (-0.27, 0.18)	-0.26 (-0.79, 0.27)	1.69 (0.48, 2.90)	0.06 (-0.35, 0.46)	0.08 (-0.82, 0.97)	3.35 (1.33, 5.37)	-0.12 (-0.37, 0.13)	-0.44 (-1.06, 0.18)	0.52 (-0.94, 1.97)
RR	0.95 (0.74, 1.20)	0.95 (0.85, 1.06)	1.06 (1.02, 1.11)	1.05 (0.74, 1.44)	1.01 (0.87, 1.17)	1.11 (1.04, 1.18)	0.85 (0.58, 1.19)	0.89 (0.74, 1.05)	1.02 (0.96, 1.09)

Q1-Q3 values are presented as ASMR per 100,000 population (95% confidence interval). COVID-19, coronavirus disease 2019; ASMR, age-standardized mortality rate; RD, rate difference; RR, rate ratio; Q1, deprivation level 1 (least); Q2, deprivation level 2; Q3, deprivation level 3 (most).

**Table 2.** Annual ASMRs and absolute and relative inequality for all-cause mortality from 2020 to 2022 by gender and region

Variables	Total			Men			Women		
	2020	2021	2022	2020	2021	2022	2020	2021	2022
Urban	310.35 (309.09, 311.61)	313.06 (311.80, 314.31)	348.67 (347.37, 349.96)	401.18 (399.03, 403.33)	402.91 (400.79, 405.03)	439.97 (437.80, 442.15)	232.77 (231.31, 234.23)	235.93 (234.47, 237.38)	269.23 (267.71, 270.74)
Deprivation level									
Lowest	300.45 (298.76, 302.14)	302.59 (300.92, 304.25)	338.41 (336.69, 340.13)	382.29 (379.42, 385.16)	382.15 (379.34, 384.96)	418.93 (416.05, 421.81)	230.59 (228.62, 232.56)	234.07 (232.12, 236.02)	268.09 (266.05, 270.13)
Middle	322.57 (320.35, 324.8)	326.12 (323.9, 328.34)	362.88 (360.58, 365.19)	420.73 (416.95, 424.5)	425.78 (422.02, 429.53)	464.33 (460.46, 468.19)	238.41 (235.84, 240.97)	240.41 (237.85, 242.97)	274.61 (271.94, 277.28)
Highest	329.28 (325.29, 333.27)	335.04 (330.97, 339.10)	366.38 (362.21, 370.55)	442.74 (435.91, 449.56)	446.10 (439.23, 452.96)	481.02 (473.99, 488.05)	231.16 (226.70, 235.61)	239.14 (234.50, 243.79)	265.92 (261.16, 270.68)
Inequality									
RD	28.83 (23.15, 34.52)	32.45 (26.72, 38.18)	27.97 (22.08, 33.86)	60.45 (50.75, 70.15)	63.95 (54.27, 73.63)	62.08 (52.18, 71.99)	0.57 (-5.86, 6.99)	5.07 (-1.52, 11.67)	-2.17 (-8.97, 4.63)
RR	1.10 (1.08, 1.12)	1.11 (1.09, 1.13)	1.08 (1.06, 1.10)	1.16 (1.13, 1.18)	1.17 (1.14, 1.19)	1.15 (1.12, 1.17)	1.00 (0.97, 1.03)	1.02 (0.99, 1.05)	0.99 (0.97, 1.02)
SII	43.07 (30.95, 55.19)	48.14 (39.08, 57.21)	42.64 (21.52, 63.76)	88.24 (87.96, 88.52)	94.06 (84.45 103.66)	92.20 (73.60, 110.80)	2.81 (-21.20, 26.82)	8.26 (-1.67, 18.18)	-1.02 (-26.54, 24.50)
RII	0.14 (0.10, 0.17)	0.15 (0.12, 0.18)	0.12 (0.06, 0.18)	0.21 (0.21, 0.21)	0.23 (0.20, 0.25)	0.20 (0.16, 0.24)	0.01 (-0.09, 0.11)	0.03 (-0.01, 0.08)	0.00 (-0.10, 0.09)
Rural	361.95 (357.76, 366.14)	360.46 (356.26, 364.66)	401.80 (397.48, 406.11)	477.38 (470.51, 484.24)	476.06 (469.17, 482.95)	511.73 (504.74, 518.73)	258.84 (253.96, 263.72)	256.03 (251.20, 260.86)	301.41 (296.33, 306.49)
Deprivation level									
Lowest	340.31 (328.85, 351.78)	335.07 (323.94, 346.19)	365.79 (354.49, 377.09)	405.54 (386.53, 424.56)	425.33 (406.38, 444.28)	443.85 (425.00, 462.70)	278.60 (264.96, 292.23)	253.96 (241.25, 266.67)	295.58 (282.22, 308.95)
Middle	359.55 (351.88, 367.21)	357.78 (350.07, 365.49)	395.29 (387.45, 403.13)	464.31 (451.83, 476.79)	460.71 (448.14, 473.28)	499.65 (486.88, 512.41)	263.70 (254.68, 272.73)	260.99 (252.02, 269.96)	295.62 (286.56, 304.68)
Highest	373.29 (367.31, 379.27)	372.81 (366.79, 378.83)	418.20 (411.94, 424.47)	500.41 (490.81, 510.00)	497.25 (487.58, 506.93)	534.31 (524.45, 544.17)	258.71 (251.67, 265.76)	259.99 (252.93, 267.04)	312.35 (304.67, 320.04)
Inequality									
RD	32.98 (15.54, 50.42)	37.74 (20.59, 54.89)	52.42 (34.85, 69.98)	94.86 (66.24, 123.48)	71.92 (43.30, 100.55)	90.46 (61.74, 119.17)	-19.88 (-40.56, 0.80)	6.03 (-13.73, 25.79)	16.77 (-4.28, 37.83)
RR	1.10 (1.04, 1.15)	1.11 (1.06, 1.17)	1.14 (1.09, 1.20)	1.23 (1.16, 1.32)	1.17 (1.10, 1.25)	1.20 (1.13, 1.28)	0.93 (0.86, 1.00)	1.02 (0.95, 1.11)	1.06 (0.99, 1.13)
SII	47.62 (12.29, 82.95)	54.20 (11.57, 96.82)	76.19 (23.62, 128.76)	134.66 (22.71, 246.60)	106.75 (53.23, 160.27)	128.85 (23.93, 233.77)	-27.31 (-60.00, 5.38)	7.05 (-11.32, 25.43)	28.97 (14.50, 43.43)
RII	0.13 (0.03, 0.23)	0.15 (0.03, 0.27)	0.19 (0.06, 0.33)	0.29 (0.05, 0.54)	0.23 (0.12, 0.35)	0.26 (0.05, 0.47)	-0.10 (-0.22, 0.02)	0.03 (-0.04, 0.10)	0.10 (0.05, 0.14)

Q1-Q3 values are presented as ASMR per 100,000 population (95% confidence interval). ASMR, age-standardized mortality rate; RD, rate difference; RR, rate ratio; Q1, deprivation level 1 (least); Q2, deprivation level 2; Q3, deprivation level 3 (most); RII, relative index of inequality; SII, slope index of inequality.

**Table 3.** Annual ASMRs and absolute and relative inequality for pneumonia-specific mortality from 2020 to 2022 by gender and region

Variables	Total			Men			Women		
	2020	2021	2022	2020	2021	2022	2020	2021	2022
Urban	18.95 (18.66, 19.23)	18.83 (18.55, 19.1)	21.03 (20.74, 21.32)	26.31 (25.79, 26.83)	26.23 (25.72, 26.74)	28.29 (27.77, 28.8)	13.65 (13.34, 13.95)	13.48 (13.18, 13.78)	15.63 (15.31, 15.95)
Deprivation level									
Lowest	18.82 (18.43, 19.22)	18.86 (18.47, 19.24)	21.31 (20.91, 21.71)	25.96 (25.23, 26.69)	25.95 (25.24, 26.66)	28.08 (27.36, 28.79)	13.71 (13.28, 14.14)	13.74 (13.31, 14.16)	16.28 (15.83, 16.74)
Middle	19.20 (18.73, 19.67)	18.87 (18.41, 19.34)	20.97 (20.49, 21.45)	26.62 (25.75, 27.49)	26.55 (25.70, 27.4)	28.78 (27.90, 29.65)	13.84 (13.32, 14.35)	13.30 (12.81, 13.79)	15.13 (14.6, 15.65)
Highest	19.14 (18.33, 19.96)	18.79 (18.00, 19.58)	20.46 (19.64, 21.27)	27.39 (25.88, 28.9)	26.86 (25.38, 28.34)	28.28 (26.8, 29.76)	13.16 (12.28, 14.04)	12.92 (12.10, 13.74)	14.67 (13.78, 15.56)
Inequality									
RD	0.32 (-0.89, 1.53)	-0.07 (-1.24, 1.11)	-0.85 (-2.07, 0.36)	1.43 (-0.81, 3.67)	0.90 (-1.29, 3.09)	0.20 (-2.00, 2.40)	-0.55 (-1.86, 0.76)	-0.81 (-2.06, 0.43)	-1.61 (-2.96, -0.27)
RR	1.02 (0.95, 1.08)	1.00 (0.94, 1.06)	0.96 (0.90, 1.02)	1.06 (0.97, 1.15)	1.03 (0.95, 1.12)	1.01 (0.93, 1.09)	0.96 (0.87, 1.06)	0.94 (0.85, 1.03)	0.90 (0.82, 0.98)
SII	0.52 (-0.05, 1.08)	-0.08 (-0.27, 0.11)	-1.19 (-1.88, -0.51)	2.02 (1.24, 2.81)	1.32 (1.25, 1.40)	0.45 (-1.39, 2.29)	-0.68 (-2.21, 0.85)	-1.17 (-1.44, -0.90)	-2.40 (-2.77, -2.04)
RII	0.03 (-0.00, 0.01)	0.00 (-0.01, 0.01)	-0.06 (-0.09, -0.02)	0.08 (0.05, 0.11)	0.05 (0.05, 0.05)	0.02 (-0.05, 0.08)	-0.05 (-0.16, 0.06)	-0.09 (-0.11, -0.07)	-0.16 (-0.18, -0.13)
Rural	24.89 (24.04, 25.73)	24.43 (23.58, 25.27)	28.28 (27.38, 29.18)	34.41 (32.91, 35.91)	34.67 (33.13, 36.21)	37.23 (35.69, 38.78)	18.50 (17.49, 19.51)	17.23 (16.34, 18.12)	21.86 (20.79, 22.94)
Deprivation level									
Lowest	18.12 (15.65, 20.59)	22.29 (19.61, 24.97)	22.89 (20.26, 25.52)	22.60 (18.22, 26.98)	32.22 (27.14, 37.29)	29.48 (24.80, 34.17)	14.65 (11.81, 17.48)	15.27 (12.44, 18.10)	17.82 (14.85, 20.79)
Middle	26.21 (24.54, 27.88)	23.83 (22.24, 25.42)	29.42 (27.71, 31.13)	35.20 (32.27, 38.13)	32.20 (29.40, 35.00)	37.52 (34.60, 40.44)	19.46 (17.55, 21.38)	17.60 (15.81, 19.39)	23.43 (21.39, 25.47)
Highest	25.30 (24.17, 26.43)	25.05 (23.91, 26.20)	28.87 (27.61, 30.12)	35.36 (33.44, 37.28)	36.17 (34.09, 38.25)	38.34 (36.28, 40.39)	18.95 (17.47, 20.43)	17.31 (16.17, 18.46)	22.19 (20.57, 23.81)
Inequality									
RD	7.18 (3.59, 10.78)	2.77 (-1.06, 6.59)	5.98 (2.09, 9.87)	12.76 (6.46, 19.05)	3.95 (-3.21, 11.10)	8.86 (2.11, 15.60)	4.30 (-0.01, 8.62)	2.04 (-1.94, 6.02)	4.37 (-0.22, 8.96)
RR	1.40 (1.17, 1.69)	1.12 (0.96, 1.34)	1.26 (1.08, 1.49)	1.56 (1.24, 2.05)	1.12 (0.91, 1.41)	1.30 (1.06, 1.63)	1.29 (1.00, 1.73)	1.13 (0.89, 1.48)	1.25 (0.99, 1.60)
SII	8.26 (-12.66, 29.19)	4.01 (1.15, 6.87)	7.07 (-9.50, 23.65)	15.51 (-15.63, 46.65)	6.82 (3.40, 10.24)	11.26 (-7.69, 30.22)	4.98 (-7.51 17.48)	2.36 (-3.75, 8.46)	4.75 (-10.24, 19.73)
RII	0.36 (-0.55, 1.26)	0.17 (0.05, 0.29)	0.26 (-0.35, 0.87)	0.50 (-0.50, 1.50)	0.20 (0.10, 0.31)	0.32 (-0.22, 0.86)	0.28 (-0.42, 0.99)	0.14 (-0.22, 0.51)	0.22 (-0.48, 0.93)

Q1-Q3 are presented as ASMR per 100,000 population (95% confidence interval). ASMR, age-standardized mortality rate; RD, rate difference; RR, rate ratio; Q1, deprivation level 1 (least); Q2, deprivation level 2; Q3, deprivation level 3 (most); RII, relative index of inequality; SII, slope index of inequality.

**Table 4.** Annual ASMRs and absolute and relative inequality for COVID-19-specific mortality from 2020 to 2022 by gender and region

Variables	Total			Men			Women		
	2020	2021	2022	2020	2021	2022	2020	2021	2022
Urban	0.92 (0.86, 0.99)	5.12 (4.97, 5.28)	26.63 (26.3, 26.96)	1.18 (1.07, 1.29)	6.54 (6.28, 6.80)	31.06 (30.51, 31.61)	0.72 (0.65, 0.80)	3.94 (3.76, 4.12)	22.91 (22.51, 23.31)
Deprivation level									
Lowest	0.95 (0.86, 1.04)	4.95 (4.75, 5.16)	26.07 (25.62, 26.52)	1.19 (1.03, 1.35)	6.12 (5.77, 6.47)	30.24 (29.49, 31)	0.76 (0.66, 0.86)	3.93 (3.69, 4.17)	22.59 (22.05, 23.14)
Middle	0.81 (0.71, 0.92)	4.91 (4.65, 5.16)	27.18 (26.62, 27.74)	1.03 (0.85, 1.20)	6.36 (5.92, 6.80)	31.62 (30.69, 32.55)	0.64 (0.53, 0.76)	3.70 (3.41, 4.00)	23.37 (22.69, 24.05)
Highest	1.18 (0.98, 1.39)	6.65 (6.14, 7.16)	27.95 (26.95, 28.96)	1.65 (1.27, 2.03)	8.95 (8.06, 9.83)	33.69 (32.01, 35.37)	0.83 (0.61, 1.05)	4.85 (4.25, 5.44)	23.25 (22.04, 24.45)
Inequality									
RD	0.23 (-0.07, 0.53)	1.69 (0.98, 2.41)	1.88 (0.42, 3.34)	0.46 (-0.07, 1.00)	2.83 (1.59, 4.06)	3.45 (1.01, 5.88)	0.07 (-0.25, 0.39)	0.92 (0.08, 1.75)	0.65 (-1.10, 2.41)
RR	1.24 (0.94, 1.61)	1.34 (1.19, 1.51)	1.07 (1.02, 1.13)	1.39 (0.95, 1.96)	1.46 (1.25, 1.70)	1.11 (1.03, 1.20)	1.09 (0.71, 1.59)	1.23 (1.02, 1.47)	1.03 (0.95, 1.11)
SII	0.27 (-0.69, 1.24)	2.25 (-1.40, 5.91)	2.72 (2.44, 3.00)	0.59 (-0.95, 2.12)	3.86 (-1.14, 8.85)	4.84 (2.20, 7.47)	0.06 (-0.48, 0.59)	1.16 (-1.51, 3.82)	1.04 (-0.12, 2.21)
RII	0.28 (-0.70, 1.26)	0.41 (-0.26, 1.07)	0.10 (0.09, 0.11)	0.48 (-0.73, 1.64)	0.54 (-0.16, 1.24)	0.15 (0.07, 0.23)	0.08 (-0.64, 0.79)	0.28 (-0.36, 0.92)	0.05 (-0.01, 0.10)
Rural	0.65 (0.51, 0.78)	2.47 (2.20, 2.74)	27.79 (26.84, 28.73)	0.75 (0.53, 0.97)	3.08 (2.63, 3.53)	33.72 (32.18, 35.26)	0.56 (0.38, 0.75)	1.92 (1.60, 2.24)	23.16 (21.97, 24.34)
Deprivation level									
Lowest	1.54 (0.84, 2.23)	1.97 (1.17, 2.76)	27.93 (24.98, 30.88)	1.72 (0.53, 2.91)	2.06 (0.78, 3.34)	33.81 (28.79, 38.83)	1.35 (0.55, 2.15)	1.74 (0.82, 2.66)	22.77 (19.33, 26.21)
Middle	0.49 (0.25, 0.74)	3.47 (2.84, 4.09)	27.95 (26.16, 29.74)	0.42 (0.10, 0.73)	4.55 (3.53, 5.58)	33.79 (30.96, 36.62)	0.58 (0.18, 0.99)	2.58 (1.78, 3.39)	23.32 (20.96, 25.68)
Highest	0.63 (0.46, 0.81)	2.13 (1.82, 2.44)	27.90 (26.6, 29.21)	0.80 (0.51, 1.08)	2.60 (2.06, 3.14)	33.87 (31.76, 35.98)	0.50 (0.27, 0.73)	1.66 (1.35, 1.97)	23.29 (21.67, 24.91)
Inequality									
RD	-0.90 (-1.77, -0.03)	0.16 (-0.94, 1.27)	-0.03 (-4.28, 4.23)	-0.92 (-2.40, 0.56)	0.54 (-1.28, 2.36)	0.06 (-7.08, 7.19)	-0.85 (-1.88, 0.18)	-0.07 (-1.31, 1.16)	0.52 (-4.54, 5.58)
RR	0.41 (0.21, 0.96)	1.08 (0.66, 2.09)	1.00 (0.86, 1.17)	0.46 (0.18, 2.06)	1.26 (0.62, 4.02)	1.00 (0.82, 1.25)	0.37 (0.13, 1.32)	0.96 (0.51, 2.42)	1.02 (0.83, 1.29)
SII	-1.20 (-4.02, 1.61)	-0.29 (-4.91, 4.33)	-0.05 (-0.14, 0.03)	-1.10 (-4.97, 2.77)	0.03 (-7.14, 7.20)	0.11 (-0.02, 0.24)	-1.23 (-3.02, 0.56)	-0.48 (-3.31, 2.36)	0.63 (-0.78, 2.04)
RII	-1.36 (-4.52, 1.81)	-0.12 (-1.95, 1.72)	0.00 (-0.00, 0.00)	-1.13 (-5.08, 2.83)	0.01 (-2.33, 2.35)	0.00 (-0.00, 0.01)	-1.51 (-3.72, 0.70)	-0.24 (-1.66, 1.18)	0.03 (-0.03, 0.09)

Q1-Q3 values are presented as ASMR per 100,000 population (95% confidence interval). ASMR, age-standardized mortality rate; COVID-19, coronavirus disease 2019; RD, rate difference; RR, rate ratio; Q1, deprivation level 1 (least); Q2, deprivation level 2; Q3, deprivation level 3 (most); RII, relative index of inequality; SII, slope index of inequality.

## Supplementary materials

Supplementary Material 1. 2020 Resident Registration Mid-Year Population of Korea.

Supplementary Material 2. 2021 Resident Registration Mid-Year Population of Korea.

Supplementary Material 3. 2022 Resident Registration Mid-Year Population of Korea.
