# Supplementary material for: Inequality in mortality according to regional deprivation during the COVID-19 pandemic
Source: Epidemiol Health. 2025 Apr 29;47:e2025022. doi: 10.4178/epih.e2025022 (PMC12425694; doi:10.4178/epih.e2025022)
Supplement: Supplementary Material 1. — 2020 Resident Registration Mid-Year Population of Korea. [file epih-47-e2025022-Supplementary-1.docx]

SUPPLEMENTARY MATERIALS

Supplementary Material 1. 2020 Resident Registration Mid-Year Population of Korea.

|  | *Total* | | | *Deprivation level* | | | | | | | | |
| --- | --- | --- | --- | --- | --- | --- | --- | --- | --- | --- | --- | --- |
|  |  |  |  | *Lowest* | | | *Middle* | | | *Highest* | | |
|  | *Men* | *Women* | *Total* | *Men* | *Women* | *Total* | *Men* | *Women* | *Total* | *Men* | *Women* | *Total* |
| *00-04* | 903,178 | 857,459.5 | 1,760,637.5 | 558,038.5 | 529,080.5 | 1,087,119 | 260,841.5 | 248,125 | 508,966.5 | 84,298 | 80,254 | 164,552 |
| *05-14* | 2,375,527 | 2,244,845.5 | 4,620,372.5 | 1,476,846 | 1,395,520 | 2,872,366 | 677,900.5 | 642,034.5 | 1,319,935 | 220,780.5 | 207,291 | 428,071.5 |
| *15-24* | 3,035,632 | 2,787,645.5 | 5,823,277.5 | 1,764,828.5 | 1,611,143.5 | 3,375,972 | 923,702.5 | 853,659 | 1,777,361.5 | 347,101 | 322,843 | 669,944 |
| *25-34* | 3,476,622.5 | 3,159,136 | 6,635,758.5 | 1,960,022 | 1,801,498.5 | 3,761,520.5 | 1,086,571.5 | 982,417 | 2,068,988.5 | 430,029 | 375,220.5 | 805,249.5 |
| *35-44* | 3,895,872.5 | 3,754,319.5 | 7,650,192 | 2,296,266 | 2,267,294.5 | 4,563,560.5 | 1,173,673 | 1,110,438 | 2,284,111 | 425,933.5 | 376,587 | 802,520.5 |
| *45-54* | 4,408,910 | 4,303,142 | 8,712,052 | 2,489,236 | 2,502,314 | 4,991,550 | 1,361,303 | 1,308,439.5 | 2,669,742.5 | 558,371 | 492,388.5 | 1,050,759.5 |
| *55-64* | 3,994,384.5 | 4,017,910 | 8,012,294.5 | 2,077,038.5 | 2,090,382 | 4,167,420.5 | 1,300,413.5 | 1,323,268 | 2,623,681.5 | 616,932.5 | 604,260 | 1,221,192.5 |
| *65-74* | 2,206,059 | 2,430,241.5 | 4,636,300.5 | 1,031,545 | 1,124,936 | 2,156,481 | 762,930.5 | 849,466 | 1,612,396.5 | 411,583.5 | 455,839.5 | 867,423 |
| *75+* | 1,309,895 | 2,188,479 | 3,498,374 | 551,606 | 907,035.5 | 1,458,641.5 | 469,600 | 772,063 | 1,241,663 | 288,689 | 509,380.5 | 798,069.5 |
| *Total* | 25,606,080.5 | 25,743,178.5 | 51,349,259 | 14,205,426.5 | 14,229,204.5 | 28,434,631 | 8,016,936 | 8,089,910 | 16,106,846 | 3,383,718 | 3,424,064 | 6,807,782 |
|  |  |  |  |  |  |  |  |  |  |  |  |  |
|  | *Urban* | | | *Deprivation level* | | | | | | | | |
|  |  |  |  | *Lowest* | | | *Middle* | | | *Highest* | | |
|  | *Men* | *Women* | *Total* | *Men* | *Women* | *Total* | *Men* | *Women* | *Total* | *Men* | *Women* | *Total* |
| *00-04* | 840,356 | 796,938 | 1,637,294 | 542,545.5 | 514,139.5 | 1,056,685 | 240,107 | 228,182 | 468,289 | 57,703.5 | 54,616.5 | 112,320 |
| *05-14* | 2,209,447.5 | 2,089,420.5 | 4,298,868 | 1,441,398 | 1,361,966 | 2,803,364 | 621,742.5 | 589,178.5 | 1,210,921 | 146,307 | 138,276 | 284,583 |
| *15-24* | 2,817,114.5 | 2,609,489.5 | 5,426,604 | 1,728,526.5 | 1,580,319.5 | 3,308,846 | 853,992 | 797,326.5 | 1,651,318.5 | 234,596 | 231,843.5 | 466,439.5 |
| *25-34* | 3,269,177 | 2,996,896.5 | 6,266,073.5 | 1,919,326.5 | 1,766,331 | 3,685,657.5 | 1,015,272.5 | 928,403.5 | 1,943,676 | 334,578 | 302,162 | 636,740 |
| *35-44* | 3,634,875.5 | 3,527,161.5 | 7,162,037 | 2,240,238.5 | 2,214,417.5 | 4,454,656 | 1,088,520.5 | 1,034,053.5 | 2,122,574 | 306,116.5 | 278,690.5 | 584,807 |
| *45-54* | 4,042,699 | 4,005,081 | 8,047,780 | 2,432,692 | 2,448,798.5 | 4,881,490.5 | 1,250,781.5 | 1,216,190 | 2,466,971.5 | 359,225.5 | 340,092.5 | 699,318 |
| *55-64* | 3,574,045.5 | 3,631,786 | 7,205,831.5 | 2,028,000.5 | 2,042,650 | 4,070,650.5 | 1,177,760.5 | 1,211,305 | 2,389,065.5 | 368,284.5 | 377,831 | 746,115.5 |
| *65-74* | 1,925,148 | 2,134,492.5 | 4,059,640.5 | 1,005,508 | 1,097,617 | 2,103,125 | 686,372.5 | 771,694 | 1,458,066.5 | 233,267.5 | 265,181.5 | 498,449 |
| *75+* | 1,101,758.5 | 1,803,605 | 2,905,363.5 | 538,649 | 883,831 | 1,422,480 | 415,806 | 680,774 | 1,096,580 | 147,303.5 | 239,000 | 386,303.5 |
| *Total* | 25,606,080.5 | 25,743,178.5 | 51,349,259 | 14,205,426.5 | 14,229,204.5 | 28,434,631 | 8,016,936 | 8,089,910 | 16,106,846 | 3,383,718 | 3,424,064 | 6,807,782 |
|  | *Rural* | | | *Deprivation level* | | | | | | | | |
|  |  |  |  | *Lowest* | | | *Middle* | | | *Highest* | | |
|  | *Men* | *Women* | *Total* | *Men* | *Women* | *Total* | *Men* | *Women* | *Total* | *Men* | *Women* | *Total* |
| *00-04* | 62,822 | 60,521.5 | 123,343.5 | 15,493 | 14,941 | 30,434 | 20,734.5 | 19,943 | 40,677.5 | 26,594.5 | 25,637.5 | 52,232 |
| *05-14* | 166,079.5 | 155,425 | 321,504.5 | 35,448 | 33,554 | 69,002 | 56,158 | 52,856 | 109,014 | 74,473.5 | 69,015 | 143,488.5 |
| *15-24* | 218,517.5 | 178,156 | 396,673.5 | 36,302 | 30,824 | 67,126 | 69,710.5 | 56,332.5 | 126,043 | 112,505 | 90,999.5 | 203,504.5 |
| *25-34* | 207,445.5 | 162,239.5 | 369,685 | 40,695.5 | 35,167.5 | 75,863 | 71,299 | 54,013.5 | 125,312.5 | 95,451 | 73,058.5 | 168,509.5 |
| *35-44* | 260,997 | 227,158 | 488,155 | 56,027.5 | 52,877 | 108,904.5 | 85,152.5 | 76,384.5 | 161,537 | 119,817 | 97,896.5 | 217,713.5 |
| *45-54* | 366,211 | 298,061 | 664,272 | 56,544 | 53,515.5 | 110,059.5 | 110,521.5 | 92,249.5 | 202,771 | 199,145.5 | 152,296 | 351,441.5 |
| *55-64* | 420,339 | 386,124 | 806,463 | 49,038 | 47,732 | 96,770 | 122,653 | 111,963 | 234,616 | 248,648 | 226,429 | 475,077 |
| *65-74* | 280,911 | 295,749 | 576,660 | 26,037 | 27,319 | 53,356 | 76,558 | 77,772 | 154,330 | 178,316 | 190,658 | 368,974 |
| *75+* | 208,136.5 | 384,874 | 593,010.5 | 12,957 | 23,204.5 | 36,161.5 | 53,794 | 91,289 | 145,083 | 141,385.5 | 270,380.5 | 411,766 |
| *Total* | 2,191,459 | 2,148,308 | 4,339,767 | 328,542 | 319,134.5 | 647,676.5 | 666,581 | 632,803 | 1,299,384 | 1,196,336 | 1,196,370.5 | 2,392,706.5 |
