# Supplementary material for: Inequality in mortality according to regional deprivation during the COVID-19 pandemic
Source: Epidemiol Health. 2025 Apr 29;47:e2025022. doi: 10.4178/epih.e2025022 (PMC12425694; doi:10.4178/epih.e2025022)
Supplement: Supplementary Material 2. — 2021 Resident Registration Mid-Year Population of Korea. [file epih-47-e2025022-Supplementary-2.docx]

Supplementary Material 2. 2021 Resident Registration Mid-Year Population of Korea.

|  | *Total* | | | *Deprivation level* | | | | | | | | |
| --- | --- | --- | --- | --- | --- | --- | --- | --- | --- | --- | --- | --- |
|  |  |  |  | *Lowest* | | | *Middle* | | | *Highest* | | |
|  | *Men* | *Women* | *Total* | *Men* | *Women* | *Total* | *Men* | *Women* | *Total* | *Men* | *Women* | *Total* |
| *00-04* | 822,670.5 | 780,737 | 1,603,407.5 | 511,036.5 | 484,544.5 | 995,581 | 236,535 | 224,586.5 | 461,121.5 | 75,099 | 71,606 | 146,705 |
| *05-14* | 2,366,133 | 2,240,737.5 | 4,606,870.5 | 1,482,656.5 | 1,404,414.5 | 2,887,071 | 669,493 | 635,461.5 | 1,304,954.5 | 213,983.5 | 200,861.5 | 414,845 |
| *15-24* | 2,896,815.5 | 2,673,265.5 | 5,570,081 | 1,695,102.5 | 1,553,246 | 3,248,348.5 | 877,765 | 815,708 | 1,693,473 | 323,948 | 304,311.5 | 628,259.5 |
| *25-34* | 3,511,480 | 3,180,998.5 | 6,692,478.5 | 1,989,252.5 | 1,815,351.5 | 3,804,604 | 1,094,995 | 991,255.5 | 2,086,250.5 | 427,232.5 | 374,391.5 | 801,624 |
| *35-44* | 3,832,060 | 3,683,505.5 | 7,515,565.5 | 2,278,208 | 2,238,697 | 4,516,905 | 1,145,488.5 | 1,082,183 | 2,227,671.5 | 408,363.5 | 362,625.5 | 770,989 |
| *45-54* | 4,373,419 | 4,277,030.5 | 8,650,449.5 | 2,487,919.5 | 2,503,970 | 4,991,889.5 | 1,343,413 | 1,294,744.5 | 2,638,157.5 | 542,086.5 | 478,316 | 1,020,402.5 |
| *55-64* | 4,057,404.5 | 4,059,165.5 | 8,116,570 | 2,128,132.5 | 2,137,530 | 4,265,662.5 | 1,310,913.5 | 1,324,279.5 | 2,635,193 | 618,358.5 | 597,356 | 1,215,714.5 |
| *65-74* | 2,361,393.5 | 2,595,613 | 4,957,006.5 | 1,115,626 | 1,218,164 | 2,333,790 | 811,557.5 | 901,866.5 | 1,713,424 | 434,210 | 475,582.5 | 909,792.5 |
| *75+* | 1,367,726 | 2,253,097.5 | 3,620,823.5 | 583,767 | 942,330.5 | 1,526,097.5 | 488,856.5 | 794,203.5 | 1,283,060 | 295,102.5 | 516,563.5 | 811,666 |
| *Total* | 25,589,102 | 25,744,150.5 | 51,333,252.5 | 14,271,701 | 14,298,248 | 28,569,949 | 7,979,017 | 8,064,288.5 | 16,043,305.5 | 3,338,384 | 3,381,614 | 6,719,998 |
|  | *Urban* | | | *Deprivation level* | | | | | | | | |
|  |  |  |  | *Lowest* | | | *Middle* | | | *Highest* | | |
|  | *Men* | *Women* | *Total* | *Men* | *Women* | *Total* | *Men* | *Women* | *Total* | *Men* | *Women* | *Total* |
| *00-04* | 765,739.5 | 726,080 | 1,491,819.5 | 496,749.5 | 470,864 | 967,613.5 | 217,595 | 206,465 | 424,060 | 51,395 | 48,751 | 100,146 |
| *05-14* | 2,201,998.5 | 2,086,657.5 | 4,288,656 | 1,446,589.5 | 1,370,037.5 | 2,816,627 | 613,514 | 582,612 | 1,196,126 | 141,895 | 134,008 | 275,903 |
| *15-24* | 2,690,573 | 2,506,041.5 | 5,196,614.5 | 1,659,762.5 | 1,523,166 | 3,182,928.5 | 810,905 | 761,851 | 1,572,756 | 219,905.5 | 221,024.5 | 440,930 |
| *25-34* | 3,308,336 | 3,024,372.5 | 6,332,708.5 | 1,948,070 | 1,780,155 | 3,728,225 | 1,024,110.5 | 938,202 | 1,962,312.5 | 336,155.5 | 306,015.5 | 642,171 |
| *35-44* | 3,580,987 | 3,462,454.5 | 7,043,441.5 | 2,222,529.5 | 2,186,172.5 | 4,408,702 | 1,062,382.5 | 1,007,051 | 2,069,433.5 | 296,075 | 269,231 | 565,306 |
| *45-54* | 4,014,588.5 | 3,983,353 | 7,997,941.5 | 2,430,720.5 | 2,449,545.5 | 4,880,266 | 1,233,848 | 1,202,674.5 | 2,436,522.5 | 350,020 | 331,133 | 681,153 |
| *55-64* | 3,630,090.5 | 3,671,845.5 | 7,301,936 | 2,077,263.5 | 2,088,153 | 4,165,416.5 | 1,185,933 | 1,211,151 | 2,397,084 | 366,894 | 372,541.5 | 739,435.5 |
| *65-74* | 2,061,428.5 | 2,285,343 | 4,346,771.5 | 1,086,941 | 1,188,047.5 | 2,274,988.5 | 729,031 | 819,171 | 1,548,202 | 245,456.5 | 278,124.5 | 523,581 |
| *75+* | 1,155,291.5 | 1,864,851 | 3,020,142.5 | 569,999.5 | 918,353.5 | 1,488,353 | 433,393 | 701,244.5 | 1,134,637.5 | 151,899 | 245,253 | 397,152 |
| *Total* | 23,409,033 | 23,610,998.5 | 47,020,031.5 | 13,938,625.5 | 13,974,494.5 | 27,913,120 | 7,310,712 | 7,430,422 | 14,741,134 | 2,159,695.5 | 2,206,082 | 4,365,777.5 |
|  | *Rural* | | | *Deprivation level* | | | | | | | | |
|  |  |  |  | *Lowest* | | | *Middle* | | | *Highest* | | |
|  | *Men* | *Women* | *Total* | *Men* | *Women* | *Total* | *Men* | *Women* | *Total* | *Men* | *Women* | *Total* |
| *00-04* | 56,931 | 54,657 | 111,588 | 14,287 | 13,680.5 | 27,967.5 | 18,940 | 18,121.5 | 37,061.5 | 23,704 | 22,855 | 46,559 |
| *05-14* | 164,134.5 | 154,080 | 318,214.5 | 36,067 | 34,377 | 70,444 | 55,979 | 52,849.5 | 108,828.5 | 72,088.5 | 66,853.5 | 138,942 |
| *15-24* | 206,242.5 | 167,224 | 373,466.5 | 35,340 | 30,080 | 65,420 | 66,860 | 53,857 | 120,717 | 104,042.5 | 83,287 | 187,329.5 |
| *25-34* | 203,144 | 156,626 | 359,770 | 41,182.5 | 35,196.5 | 76,379 | 70,884.5 | 53,053.5 | 123,938 | 91,077 | 68,376 | 159,453 |
| *35-44* | 251,073 | 221,051 | 472,124 | 55,678.5 | 52,524.5 | 108,203 | 83,106 | 75,132 | 158,238 | 112,288.5 | 93,394.5 | 205,683 |
| *45-54* | 358,830.5 | 293,677.5 | 652,508 | 57,199 | 54,424.5 | 111,623.5 | 109,565 | 92,070 | 201,635 | 192,066.5 | 147,183 | 339,249.5 |
| *55-64* | 427,314 | 387,320 | 814,634 | 50,869 | 49,377 | 100,246 | 124,980.5 | 113,128.5 | 238,109 | 251,464.5 | 224,814.5 | 476,279 |
| *65-74* | 299,965 | 310,270 | 610,235 | 28,685 | 30,116.5 | 58,801.5 | 82,526.5 | 82,695.5 | 165,222 | 188,753.5 | 197,458 | 386,211.5 |
| *75+* | 212,434.5 | 388,246.5 | 600,681 | 13,767.5 | 23,977 | 37,744.5 | 55,463.5 | 92,959 | 148,422.5 | 143,203.5 | 271,310.5 | 414,514 |
| *Total* | 2,180,069 | 2,133,152 | 4,313,221 | 333,075.5 | 323,753.5 | 656,829 | 668,305 | 633,866.5 | 1,302,171.5 | 1,178,688.5 | 1,175,532 | 2,354,220.5 |
