# Supplementary material for: Inequality in mortality according to regional deprivation during the COVID-19 pandemic
Source: Epidemiol Health. 2025 Apr 29;47:e2025022. doi: 10.4178/epih.e2025022 (PMC12425694; doi:10.4178/epih.e2025022)
Supplement: Supplementary Material 3. — 2022 Resident Registration Mid-Year Population of Korea. [file epih-47-e2025022-Supplementary-3.docx]

Supplementary Material 3. 2022 Resident Registration Mid-Year Population of Korea.

|  | *Total* | | | *Deprivation level* | | | | | | | | |
| --- | --- | --- | --- | --- | --- | --- | --- | --- | --- | --- | --- | --- |
|  |  |  |  | *Lowest* | | | *Middle* | | | *Highest* | | |
|  | *Men* | *Women* | *Total* | *Men* | *Women* | *Total* | *Men* | *Women* | *Total* | *Men* | *Women* | *Total* |
| *00-04* | 758,337 | 720,392 | 1,478,729 | 471,823.5 | 448,131 | 919,954.5 | 218,372 | 20,7335.5 | 425,707.5 | 68,141.5 | 64,925.5 | 133,067 |
| *05-14* | 2,322,191 | 2,201,463 | 4,523,654 | 1,463,369 | 1,387,938 | 2,851,307 | 653,633 | 621,020 | 1,274,653 | 205,189 | 192,505 | 397,694 |
| *15-24* | 2,785,531.5 | 2,579,013 | 5,364,544.5 | 1,637,528 | 1,504,090 | 3,141,618 | 841,751 | 785,306.5 | 1,627,057.5 | 306,252.5 | 289,616.5 | 595,869 |
| *25-34* | 3,540,565 | 3,204,546 | 6,745,111 | 2,011,492.5 | 1,825,363 | 3,836,855.5 | 1,102,985.5 | 1,002,642.5 | 2,105,628 | 426,087 | 376,540.5 | 802,627.5 |
| *35-44* | 3,759,949 | 3,606,217 | 7,366,166 | 2,245,960 | 2,198,545.5 | 4,444,505.5 | 1,119,911 | 1,056,835 | 2,176,746 | 394,078 | 350,836.5 | 744,914.5 |
| *45-54* | 4,331,612 | 4,241,971.5 | 8,573,583.5 | 2,482,209 | 2,498,803 | 4,981,012 | 1,324,111.5 | 1,278,895 | 2,603,006.5 | 525,291.5 | 464,273.5 | 989,565 |
| *55-64* | 4,099,591 | 4,097,226.5 | 8,196,817.5 | 2,166,054 | 2,180,188.5 | 4,346,242.5 | 1,315,803 | 1,325,975 | 2,641,778 | 617,734 | 591,063 | 1,208,797 |
| *65-74* | 2,500,757.5 | 2,735,022.5 | 5,235,780 | 1,192,538.5 | 1,299,815.5 | 2,492,354 | 854,824 | 945,348.5 | 1,800,172.5 | 453,395 | 489,858.5 | 943,253.5 |
| *75+* | 1,441,508 | 2,333,256 | 3,774,764 | 623,185.5 | 985,830.5 | 1,609,016 | 513,361.5 | 821,457 | 1,334,818.5 | 304,961 | 525,968.5 | 830,929.5 |
| *Total* | 25,540,042 | 25,719,107.5 | 51,259,149.5 | 14,294,160 | 14,328,705 | 28,622,865 | 7,944,752.5 | 8,044,815 | 15,989,567.5 | 3,301,129.5 | 3,345,587.5 | 6,646,717 |
|  | *Urban* | | | *Deprivation level* | | | | | | | | |
|  |  |  |  | *Lowest* | | | *Middle* | | | *Highest* | | |
|  | *Men* | *Women* | *Total* | *Men* | *Women* | *Total* | *Men* | *Women* | *Total* | *Men* | *Women* | *Total* |
| *00-04* | 706,696 | 670,931 | 1,377,627 | 458,806 | 435,726 | 894,532 | 201,040 | 190,747.5 | 391,787.5 | 46,850 | 44,457.5 | 91,307.5 |
| *05-14* | 2,162,238 | 2,051,261.5 | 4,213,499.5 | 1,427,336 | 1,353,507.5 | 2,780,843.5 | 598,720 | 569,201.5 | 1,167,921.5 | 136,182 | 128,552.5 | 264,734.5 |
| *15-24* | 2,589,739.5 | 2,420,306.5 | 5,010,046 | 1,603,139.5 | 1,474,675.5 | 3,077,815 | 777,526 | 733,448 | 1,510,974 | 209,074 | 212,183 | 421,257 |
| *25-34* | 3,340,633 | 3,051,148.5 | 6,391,781.5 | 1,970,120.5 | 1,790,422 | 3,760,542.5 | 1,032,313.5 | 949,789 | 1,982,102.5 | 338,199 | 310,937.5 | 649,136.5 |
| *35-44* | 3,518,176 | 3,391,766 | 6,909,942 | 2,191,465 | 2,147,066 | 4,338,531 | 1,038,995.5 | 983,518.5 | 2,022,514 | 287,715.5 | 261,181.5 | 548,897 |
| *45-54* | 3,980,125 | 3,952,729.5 | 7,932,854.5 | 2,424,499 | 2,443,902 | 4,868,401 | 1,215,613 | 1,187,170 | 2,402,783 | 340,013 | 321,657.5 | 661,670.5 |
| *55-64* | 3,667,414 | 3,708,924.5 | 7,376,338.5 | 2,113,826 | 2,129,467.5 | 4,243,293.5 | 1,189,356.5 | 1,211,876.5 | 2,401,233 | 364,231.5 | 367,580.5 | 731,812 |
| *65-74* | 2,183,103 | 2,412,699 | 4,595,802 | 1,161,531.5 | 1,267,248.5 | 2,428,780 | 766,461 | 858,108 | 1,624,569 | 255,110.5 | 287,342.5 | 542,453 |
| *75+* | 1,222,512.5 | 1,941,009 | 3,163,521.5 | 608,385.5 | 960,866.5 | 1,569,252 | 455,747 | 726,803.5 | 1,182,550.5 | 158,380 | 253,339 | 411,719 |
| *Total* | 23,370,637 | 23,600,775.5 | 46,971,412.5 | 13,959,109 | 14,002,881.5 | 27,961,990.5 | 7,275,772.5 | 7,410,662.5 | 14,686,435 | 2,135,755.5 | 2,187,231.5 | 4,322,987 |
|  | *Rural* | | | *Deprivation level* | | | | | | | | |
|  |  |  |  | *Lowest* | | | *Middle* | | | *Highest* | | |
|  | *Men* | *Women* | *Total* | *Men* | *Women* | *Total* | *Men* | *Women* | *Total* | *Men* | *Women* | *Total* |
| *00-04* | 51,641 | 49,461 | 101,102 | 13,017.5 | 12,405 | 25,422.5 | 17,332 | 16,588 | 33,920 | 21,291.5 | 20,468 | 41,759.5 |
| *05-14* | 159,953 | 150,201.5 | 310,154.5 | 36,033 | 34,430.5 | 70,463.5 | 54,913 | 51,818.5 | 106,731.5 | 69,007 | 63,952.5 | 132,959.5 |
| *15-24* | 195,792 | 158,706.5 | 354,498.5 | 34,388.5 | 29,414.5 | 63,803 | 64,225 | 51,858.5 | 116,083.5 | 97,178.5 | 77,433.5 | 174,612 |
| *25-34* | 199,932 | 153,397.5 | 353,329.5 | 41,372 | 34,941 | 76,313 | 70,672 | 52,853.5 | 123,525.5 | 87,888 | 65,603 | 153,491 |
| *35-44* | 241,773 | 214,451 | 456,224 | 54,495 | 51,479.5 | 105,974.5 | 80,915.5 | 73,316.5 | 154,232 | 106,362.5 | 89,655 | 196,017.5 |
| *45-54* | 351,487 | 289,242 | 640,729 | 57,710 | 54,901 | 112,611 | 108,498.5 | 91,725 | 200,223.5 | 185,278.5 | 142,616 | 327,894.5 |
| *55-64* | 432,177 | 388,302 | 820,479 | 52,228 | 50,721 | 102,949 | 126,446.5 | 114,098.5 | 240,545 | 253,502.5 | 223,482.5 | 476,985 |
| *65-74* | 317,654.5 | 322,323.5 | 639,978 | 31,007 | 32,567 | 63,574 | 88,363 | 87,240.5 | 175,603.5 | 198,284.5 | 202,516 | 400,800.5 |
| *75+* | 218,995.5 | 392,247 | 611,242.5 | 14,800 | 24,964 | 39,764 | 57,614.5 | 94,653.5 | 152,268 | 146,581 | 272,629.5 | 419,210.5 |
| *Total* | 2,169,405 | 2,118,332 | 4,287,737 | 335,051 | 325,823.5 | 660,874.5 | 668,980 | 634,152.5 | 1,303,132.5 | 1,165,374 | 1,158,356 | 2,323,730 |
